# Enhancement of tetragonal anisotropy and stabilisation of the tetragonal phase by Bi/Mn-double-doping in BaTiO_3_ ferroelectric ceramics

**DOI:** 10.1038/srep45842

**Published:** 2017-04-03

**Authors:** Hisato Yabuta, Hidenori Tanaka, Tatsuo Furuta, Takayuki Watanabe, Makoto Kubota, Takanori Matsuda, Toshihiro Ifuku, Yasuhiro Yoneda

**Affiliations:** 1 R&D Headquarters, Canon Inc., Ohta, Tokyo 146-8501, Japan; 2 Reaction Dynamics Research Center, Japan Atomic Energy Agency (JAEA), Sayo-cho, Hyogo 679-5148, Japan

## Abstract

To stabilise ferroelectric-tetragonal phase of BaTiO_3_, the double-doping of Bi and Mn up to 0.5 mol% was studied. Upon increasing the Bi content in BaTiO_3_:Mn:Bi, the tetragonal crystal-lattice-constants *a* and *c* shrank and elongated, respectively, resulting in an enhancement of tetragonal anisotropy, and the temperature-range of the ferroelectric tetragonal phase expanded. X-ray absorption fine structure measurements confirmed that Bi and Mn were located at the A(Ba)-site and B(Ti)-site, respectively, and Bi was markedly displaced from the centrosymmetric position in the BiO_12_ cluster. This A-site substitution of Bi also caused fluctuations of B-site atoms. Magnetic susceptibility measurements revealed a change in the Mn valence from +4 to +3 upon addition of the same molar amount of Bi as Mn, probably resulting from a compensating behaviour of the Mn at Ti^4+^ sites for donor doping of Bi^3+^ into the Ba^2+^ site. Because addition of La^3+^ instead of Bi^3+^ showed neither the enhancement of the tetragonal anisotropy nor the stabilisation of the tetragonal phase, these phenomena in BaTiO_3_:Mn:Bi were not caused by the Jahn-Teller effect of Mn^3+^ in the MnO_6_ octahedron, but caused by the Bi-displacement, probably resulting from the effect of the 6 *s* lone-pair electrons in Bi^3+^.

Barium titanate (BaTiO_3_) is one of the most well-known ferroelectric materials and has been used in electrical components such as multilayered ceramic capacitors^1^ and positive temperature coefficient thermistors^2^. BaTiO_3_ has outstanding ferroelectric and piezoelectric properties at room temperature, but shows a relatively low Curie temperature of about 130 °C and another phase transition (from tetragonal to orthorhombic) near room temperature. These phase transitions near room temperature and the narrow stable temperature range of the ferroelectric tetragonal phase are disadvantages for ferroelectric and piezoelectric applications.

To adjust the electrical characteristics of BaTiO_3_-based ceramic materials, especially to improve their insulating properties, some transition metal elements such as Mn, sometimes accompanied with non-transition metal doping elements, are often added to BaTiO_3_^1^,^3^,^4^,^5^,^6^. However, adding a small amount of Mn to BaTiO_3_ lowers its ferroelectric transition temperature (Curie temperature; *T*_C_)^7^. This decrease in *T*_C_, while favourable for capacitor applications, is still a problem for ferroelectric and piezoelectric applications. For ferroelectric and piezoelectric device development, BaTiO_3_ is often alloyed with high-*T*_C_ ferroelectric materials such as bismuth ferrite (BiFeO_3_). The resulting BaTiO_3_-BiFeO_3_ solid solution shows remarkable properties, including large field-induced strain, and unusual electro-structural behaviour such as ferroelectricity in a pseudo-cubic crystal lattice^8^,^9^. We expected that doping of Bi and transition metal elements into BaTiO_3_ would result in electrical properties superior to those of undoped one. Hence, we have investigated the effect of adding Bi to Mn-doped BaTiO_3_, which is one of the most common doped BaTiO_3_ ceramics. However, adding only 4% BiFeO_3_ to BaTiO_3_ made the ferroelectric phase transition diffuse, as in relaxor-ferroelectrics such as Pb(Mg_1/3_Nb_1/2_)O_3_-PbTiO_3_^10^. Therefore, to retain normal ferroelectric behaviour in BaTiO_3_, including sharp first-order phase transitions, we have investigated how *T*_C_ increases and how other characteristics improve with doping concentrations of less than 1%.

In the current work, we studied the effects of double-doping BaTiO_3_ with a very small amount of Bi/Mn on phase transitions, dielectric and ferroelectric properties, long-range (averaged) crystal lattice structures, short- and medium-range atomic correlations, and local structures around the doped atoms. In addition, the chemical states of the dopants were directly estimated. Finally, we discuss the microscopic mechanism of structural and electrical property evolution, and the variation of phase transition temperatures caused by low-level doping of Bi/Mn into BaTiO_3_.

## Results

### Electrical measurements

**Figure 1:**
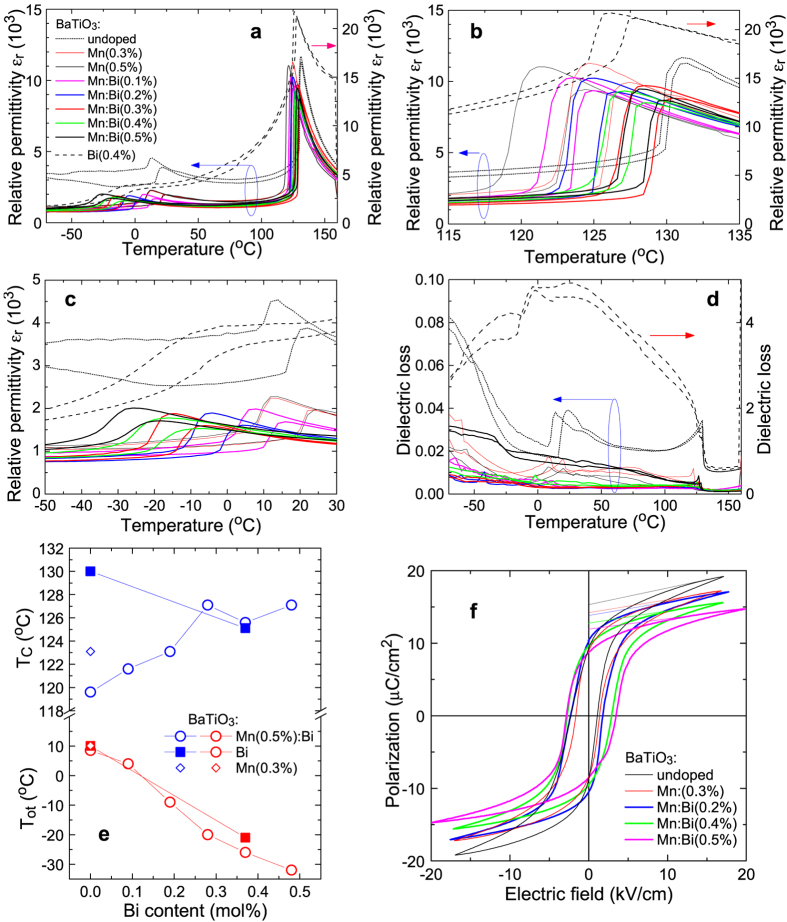
(**a**) Temperature dependence of the relative permittivity, *ε*_r_, for undoped and Bi/Mn-doped BaTiO_3_. (**b**) Expanded view of Fig. 1a around the Curie temperature, *T*_C_. (**c**) Expanded view of Fig. 1a around the orthorhombic-tetragonal phase transition temperature, *T*_ot_. (**d**) Temperature dependence of the dielectric loss (tan δ) for undoped and Bi/Mn-doped BaTiO_3_. (**e**) Dependence of *T*_C_ and *T*_ot_ on Bi concentration for Bi- and Bi/Mn-doped BaTiO_3_. (**f**) Polarisation-electric field (*P*-*E*) curves for undoped, Mn(0.3%)-doped, and Bi(0.2%,0.4%,0.5%)/Mn(0.5%)-doubly doped BaTiO_3_ with an AC field frequency of 1 Hz at room temperature. The thin straight lines are extrapolation lines of polarisations at high fields to zero field for evaluating spontaneous polarisation *P*_s_.

To characterise the effects of doping Bi/Mn into BaTiO_3_ on its electrical properties, the temperature dependence of the relative dielectric permittivity (*ε*_r_) (Fig. 1a–c) and of the dielectric loss (tan δ) (Fig. 1d) for undoped, Mn-doped, Bi-doped, and Bi/Mn-doubly doped BaTiO_3_ were measured, with the results shown at 1 kHz. The doped compositions are abbreviated as BaTiO_3_:Mn, BaTiO_3_:Bi, and BaTiO_3_:Mn:Bi, respectively, and *ε*_r_ = *ε/ε*_0_, where *ε* is the permittivity of the material and *ε*_0_ is the vacuum permittivity. Dielectric anomalies were observed at around 120 and 0 °C, which correspond to the cubic (paraelectric)-tetragonal (ferroelectric) and the tetragonal-orthorhombic phase transitions, respectively. Compared with the dielectric properties of undoped BaTiO_3_, BaTiO_3_;Mn and BaTiO_3_:Mn:Bi samples showed relatively low *ε*_r_ values below the cubic-tetragonal phase transition temperature, which is equal to *T*_C_, regardless of Bi content, and also relatively low dielectric loss. However, BaTiO_3_:Bi without Mn exhibited a large dielectric permittivity and a huge dielectric loss (Fig. 1a and d). Mn is the dopant of choice to suppress the dielectric loss factor and leakage current for BaTiO_3_-based capacitors^4^,^11^, due to compensation for unintended impurities that create mobile carriers^12^,^13^. Therefore, the reduction of dielectric loss by Mn-doping in our experiment was attributed to the same mechanism. Presumably, the relatively large permittivity values for BaTiO_3_:Bi were due to charge carriers, which induced the huge dielectric loss, and Mn-doping caused the reduction of dielectric loss together with a decrease in permittivity. The increase in the dielectric loss, namely the increase in leakage current, by Bi-doping without Mn was probably caused by the creation of carrier electrons or holes in Ba^2+^Ti^4+^O_3_ by doping of aliovalent Bi^3+^ or Bi^5+^. However, it is worth noting that Bi/Mn-doubly doped BaTiO_3_ samples showed excellent dielectric behaviour with low dielectric loss regardless of Bi content, as did BaTiO_3_:Mn. This result suggests that Mn-doping compensated for the creation of charge carriers by Bi-doping.

Bi/Mn-doping also affected the phase transition temperatures. *T*_C_ decreased with increasing amounts of Mn in BaTiO_3_:Mn, and this lowered *T*_C_ by Mn-doping increased with increasing amounts of Bi in BaTiO_3_:Mn:Bi, and then roughly recovered by 0.5 mol% of Bi (Fig. 1b). In contrast, the tetragonal-orthorhombic phase transition temperature (*T*_ot_) was not affected by doping Mn into BaTiO_3_, and decreased with increasing amounts of Bi in BaTiO_3_:Mn:Bi (Fig. 1c). Doping of Bi into BaTiO_3_ without Mn caused both phase transitions to broaden and the transition temperatures to decrease. Adding Bi to BaTiO_3_:Mn increased *T*_C_ and decreased *T*_ot_; that is, the addition of Bi stabilised the tetragonal phase by expanding its temperature range (Fig. 1e).

Figure 1f shows the polarisation-electric field (*P*-*E*) curves for undoped, Mn(0.3%)-doped, and Bi(0.2–0.5%)/Mn(0.5%)-doubly doped BaTiO_3_. The *P*-*E* curves indicate undoped BaTiO_3_ yielded a larger spontaneous polarisation (*P*_s_), which can be evaluated from extrapolation of saturated polarisation at high fields to zero field^14^ as indicated with thin straight lines in the figure, than did the other doped ones. Mn-doping into BaTiO_3_ decreased *P*_s_, and Bi-doping into BaTiO_3_:Mn decreased *P*_s_ further. Increasing the amount of Bi doped into BaTiO_3_:Mn increased the coercive field (*E*_c_), probably by impeding domain switching. This effect of Bi-doping was observed only in Bi/Mn-doubly doped BaTiO_3_ because BaTiO_3_:Bi showed a leakage current that was too large to carry out the *P*-*E* measurement, owing to the charge carriers created by Bi-doping.

### Rietveld analysis from X-ray powder diffraction

To determine the detailed lattice structures of Bi/Mn-doped BaTiO_3_, powder diffraction measurements at room temperature with a synchrotron X-ray source was carried out. All BaTiO_3_-based samples had a PbTiO_3_-type tetragonal crystal structure (space group: *P*4*mm*).

**Figure 2:**
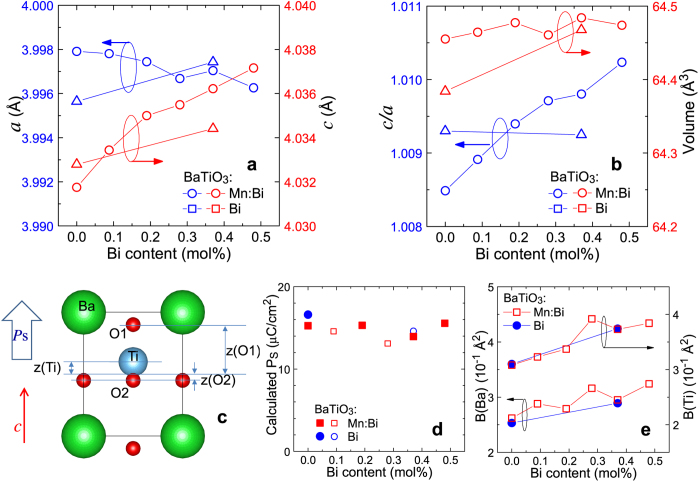
(**a**) Lattice constants *a* and *c* of Bi-doped and Bi/Mn-doubly doped BaTiO_3_ as a function of Bi content. (**b**) Ratio of lattice constants *c/a* and lattice volume of Bi-doped and Bi/Mn-doubly doped BaTiO_3_ as a function of Bi content. (**c**) Schematic view of the ferroelectric BaTiO_3_ crystal lattice, with *c*-axis atomic positions of Ti and O, *z*(Ti), *z*(O1), and *z*(O2), related to spontaneous polarization *P*_s_. (**d**) *P*_s_ values calculated from the results of Rietveld analysis for Bi-doped and Bi/Mn-doubly doped BaTiO_3_ as a function of Bi content. Plotted points for the samples indicated in Fig. 1f are highlighted as larger closed symbols. (**e**) Atomic displacement factors of Ba and Ti (denoted as *B*(Ba) with left axis and *B*(Ti) with right axis, respectively) in Bi-doped and Bi/Mn-doubly doped BaTiO_3_ as a function of Bi content.

Figure 2a shows the tetragonal lattice constants *a* and *c* estimated by the first Rietveld analysis, which is only for determining the lattice constants using diffraction data from a sample mixed with a standard (details are shown in Supplementary Information). The lattice parameter ratio, *c/a*, and unit cell volume (=*a*^2^*c*) are also shown in Fig. 2b. The effect of doping Mn into BaTiO_3_ on the lattice can be seen in the figure by comparing the values for BaTiO_3_:Mn (triangle) and undopd BaTiO_3_ (circle) at a Bi content of zero. When doped with Mn, the BaTiO_3_ lattice elongated in the *a* direction but slightly shrank in the *c* direction, resulting in a decrease in the *c/a* ratio and an increase in the unit cell volume. Bi/Mn-doubly doped BaTiO_3_ exhibited a slight decrease in *a* and increase in *c* with increasing Bi content, resulting in an increase in the *c/a* ratio and an almost unchanged unit cell volume. These results indicate that doping BaTiO_3_ with Mn suppressed the anisotropy of the tetragonal crystal lattice and expanded the lattice, and then further doping of BaTiO_3_:Mn with Bi increased the anisotropy with no effect on volume. In contrast, doping BaTiO_3_ with Bi and without Mn yielded increases in both *a* and *c*, resulting in a simple lattice expansion without a change in the *c/a* ratio, preserving the anisotropy of the lattice. These results suggest that doping Bi into BaTiO_3_ enhances its tetragonal anisotropy only in the presence of Mn.

Atomic positions in the tetragonal lattice with space group *P*4*mm* were estimated by applying the second Rietveld analysis using the lattice constants obtained from the first analysis (details are shown in Supplementary Information). In this symmetry shown in Fig. 2c, the positions of the Ti, O1 and O2 atoms along the *c*-axis, denoted as *z*(Ti), *z*(O1), and *z*(O2), respectively, are the variable parameters if the Ba position is fixed, and the deviation of these positions from the centrosymmetrical positions, at *z*(Ti) =*z* (O2) = 0 and *z*(O1) = 1/2, should be related to spontaneous polarisation, *P*_s_^15^, which is expressed with a classical point charge model^14^ as


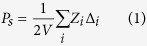


Here *V* is the unit-cell volume, and *Z*_*i*_ and *Δ*_*i*_ are the effective charges and atomic displacement vectors, respectively, for the *i*th ions. *P*_s_ values calculated from the results of Rietveld analysis with the assumption of the point charge model equation (1) are shown in Fig. 2d. The magnitude of *P*_s_ for each sample was calculated to be about 18 μC/cm^2^, and the *P*_s_ values of Mn-doped and Bi/Mn-doubly doped BaTiO_3_ were slightly smaller than that of undoped BaTiO_3_, consistent with the *P*-*E* results shown in Fig. 1f.

The atomic displacement factors of the Ba and Ti sites estimated by the second Rietveld analysis, denoted as *B*(Ba) and *B*(Ti), respectively, are shown in Fig. 2e. Both *B*(Ba) and *B*(Ti) increased with increasing Bi content. As described in the next subsection, doped Bi was located at the Ba site (“A site”) and Mn at the Ti site (“B site”). Doping with Bi was expected to cause *B*(Ba) to increase because the randomness of A-site atoms would be increased by the substitution of Bi for Ba. However, the increase of *B*(Ti) upon substitution of the A site with a small amount of Bi is unusual, and may have resulted from some indirect effect of A-site-substituted Bi on the B-site atoms. The parameters obtained by the Rietveld analyses are provided in Supplementary Information.

### X-ray absorption fine structure (XAFS)

**Figure 3:**
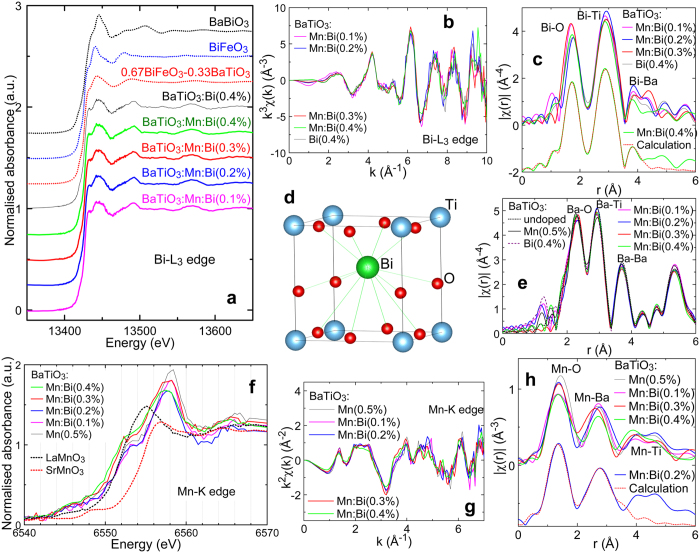
(**a**) Bi-*L*_3_ XANES spectra of Bi-doped and Bi/Mn-doubly doped BaTiO_3_, and of standards BaBiO_3_, BiFeO_3_, and 0.67BiFeO_3_-0.33BaTiO_3_ solid solution. (**b**) Bi-*L*_3_ EXAFS spectra *k*^3^*χ(k*) of Bi-doped and Bi/Mn-doubly doped BaTiO_3_. (**c**) RSFs for Bi-doped and Bi/Mn-doubly doped BaTiO_3_ from Bi-*L*_3_ EXAFS spectra, and analytical results of the Bi RSF of BaTiO_3_:Mn:Bi(0.4%). (**d**) Schematic view of Bi and O displacements with respect to Ti atom coordination. (**e**) RSFs for undoped, Mn-doped, Bi-doped, and Bi/Mn-doubly doped BaTiO_3_ from Ba-*K* EXAFS spectra. (**f**) Mn-*K* XANES spectra of Mn-doped and Bi/Mn-doubly doped BaTiO_3_, and LaMnO_3_ and SrMnO_3_ standards. (**g**) Mn EXAFS spectra *k*^2^*χ(k*) of Mn-doped and Bi/Mn-doubly doped BaTiO_3_. (**h**) RSFs for Mn-doped and Bi/Mn-doubly doped BaTiO_3_ from Mn-*K* EXAFS spectra, and analytical results of the Mn RSF of BaTiO_3_:Mn:Bi(0.2%).

Figure 3a shows the Bi-*L*_3_ X-ray absorption near edge structure (XANES) spectra of BaTiO_3_:Bi and BaTiO_3_:Mn:Bi samples, and of BaBiO_3_, BiFeO_3_, and 0.67BiFeO_3_-0.33BaTiO_3_ solid solution standards. The Bi-*L*_3_ edge energies for BaTiO_3_:Bi and BaTiO_3_:Mn:Bi were similar to those of BiFeO_3_ and 0.67BiFeO_3_-0.33BaTiO_3_, in which the Bi valence is +3, although the edge energies were different from that of BaBiO_3_ with a mixed Bi^3+^/Bi^5+^ valence state. In addition, the oscillation structures of the Bi-*L*_3_ XANES spectra of the BaTiO_3_-based materials were similar to those of the BiFeO_3_-based substances, especially that of pseudo-cubic 0.67BiFeO_3_-0.33BaTiO_3_, but different from that of BaBiO_3_. Bi atoms are located at the A sites of perovskite-type structures in distorted rhombohedral BiFeO_3_^16^ and pseudo-cubic 0.67BiFeO_3_-0.33BaTiO_3_^17^, whereas they are located at B sites in BaBiO_3_^18^. Therefore, Bi in the BaTiO_3_:Bi and BaTiO_3_:Mn:Bi samples had a valence state of +3 and were located at the A site in the perovskite-type structure, and thus at the Ba site in tetragonal BaTiO_3_ with a *c/a* ratio close to 1.

Bi-*L*_3_ extended X-ray absorption fine structure (EXAFS) spectra *χ(k*) for BaTiO_3_:Bi and BaTiO_3_:Mn:Bi samples are shown in Fig. 3b with a *k*-weighted form of *k*^3^*χ(k*) as a function of wavenumber *k*. All the samples yielded spectra with very similar profiles between 2 and 10 Å^−1^, regardless of the amount of Bi doping and whether Mn was present. The Bi radial structure function (RSF) of each sample was derived by the Fourier transform of *k*^3^*χ(k*), and all the RSFs are shown in Fig. 3c. As expected from the Bi-*L*_3_ EXAFS spectra of Fig. 3b, the RSFs were similar to each other, suggesting similar local structures around the Bi atom in all the samples. To determine the local structure around the Bi atom, curve-fitting analysis of the RSF was carried out by using the single-scattering EXAFS equation^19^,^20^,^21^


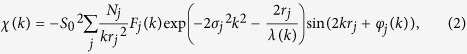


where *r*_*j*_ is the distance from the central absorbing atom to the *j*th shell atoms, *N*_*j*_ is the coordination number, *σ*^2^_*j*_ is the Debye-Waller factor, *F*_*j*_(*k*) is the complex backscattering amplitude, *φ*_*j*_(*k*) is the phase shift of the atoms in the *j*th shell, *S*_0_ is the overall amplitude reduction factor due to the many-body effect (so-called *intrinsic loss* factor), and *λ(k*) is the mean-free path of the photoelectron (e^−2*r*/λ^ is so-called *extrinsic loss* factor related to inelastic scattering). In this analysis, we employed *r*_*j*_, *σ*^2^_*j*_, and *ΔE*_0_ which is the correction of the absorption edge energy, as the variables to refine. *S*_0_ was fixed as 1.09, which was derived from EXAFS analysis of the BiFeO_3_ standard sample, imposing constraints based on the symmetry of the crystal structure^22^. *F*_*j*_(*k*), λ_*j*_(*k*), and λ(*k*) were calculated by using FEFF in ARTEMIS software and then these parameters were applied to the EXAFS analysis automatically. These RSFs did not fit well with a structural model of Bi at the Ba site based on the Ba local structure in the BaTiO_3_ crystal derived from the Rietveld analysis. The obtained reliability factor, R, which indicates quality of fit, from a fit with this model was on the order of 10^−2^. However, fitting was drastically improved (R reduced to ~10^−4^) by using a model with off-centre Bi compared with the ideal Ba position in the BaTiO_3_ crystal lattice. The fitting results were not sensitive to the direction of Bi displacement, although they were sensitive to the difference in *r* between shorter and longer Bi-O bonds, and also the difference of *r* for different Bi-Ti bonds. Therefore, we assumed that Bi was displaced mainly along a slightly elongated *c*-axis in the tetragonal lattice, like the model used to analyse the local Pb structure in highly distorted tetragonal PbTiO_3_^23^. Representative fitting results for the RSFs of BaTiO_3_:Bi and BaTiO_3_:Mn:Bi(0.4%) shown in Fig. 3c are listed in Table 1, and a comparison of the fitted curve to an experimentally obtained RSF of BaTiO_3_:Mn:Bi(0.4%) is shown at a the bottom of Fig. 3c. The calculated curve fits the RSF well, and the R-factors are very small, meaning that the off-centre Bi structural model reproduced the RSFs of BaTiO_3_:Bi and BaTiO_3_:Mn:Bi. Figure 3d illustrates the local structure around Bi with the parameters of BaTiO_3_:Mn:Bi(0.4%) in Table 1. The large displacement of Bi from the centrosymmetric position and a distorted BiO_12_ cluster are visible. The average length of Bi-O bonds is much shorter than the Ba-O bonds calculated from the Rietveld analysis. This difference is caused by the substitution of the higher-valence Bi^3+^ for Ba^2+^, explained by the electrostatic force between the cations and O^2−^; in contrast, the average length of the Bi-Ti bonds is almost unchanged from that of Ba-Ti. Doping the BaTiO_3_-based crystal with a very small amount of Bi yielded a local Bi displacement and shrinkage of Bi-O bonds, which appeared to have affected the entire crystal, especially the length of the *c*-axis, according to the results of Rietveld analysis (Fig. 2a).

Figure 3e shows Ba RSFs for the undoped and doped BaTiO_3_ samples from Ba-*K* EXAFS spectra *k*^3^*χ(k*). All of these RSFs were very similar, indicating that the local structure around the Ba atom in Mn-doped, Bi-doped, and Bi/Mn-doubly doped BaTiO_3_ was almost unchanged from that of undoped BaTiO_3_. However, there was a slight decrease in RSF intensity, especially for the Ba-Ba peak, which suggests a small increase in the Debye-Waller factor of Ba, and which is consistent with the result of the Rietveld analysis shown in Fig. 2e.

Mn-*K* XANES spectra of BaTiO_3_:Mn, and BaTiO_3_:Mn:Bi, and the LaMn^3+^O_3_ and SrMn^4+^O_3_ standards are shown in Fig. 3f. The Mn-*K* absorption edge and the white-line peak beside the edge shifted slightly from higher energy to lower energy as the amount of doped Bi was increased. Similarly, the edge of the AMnO_3_ perovskite-type materials (A = Sr or La) shifted from higher energy for SrMn^4+^O_3_, which has a relatively high Mn valence, to lower energy for LaMn^3+^O_3_ with its lower Mn valence. This result suggests that Mn valence in BaTiO_3_:Mn:Bi may decrease with increasing Bi content.

The various BaTiO_3_:Mn and BaTiO_3_:Mn:Bi samples yielded very similar Mn-*K* EXAFS spectra *k*^2^*χ(k*) (Fig. 3g), suggesting that the doped Mn atoms were in the same location and had similar local structures in these samples, regardless of the amount of Bi. Because the Mn-fluorescent XAFS signals were weak, the *k*^2^*χ(k*) profiles in high *k* region were noisy, so that only a *k* range of 2 to 7 Å^−1^ could be used for the RSF calculations. The calculated Mn RSFs in Fig. 3h are similar, as expected from the *k*^2^*χ(k*) profiles, but with small differences in the details, probably due to the noisy *k*^2^*χ(k*) spectra. Therefore, the quantitative description provided above for the Bi-*L*_3_ EXAFS analysis appears to not be appropriate for the EXAFS analysis with these Mn RSFs, but these RSFs can nevertheless be used to qualitatively check substitution sites in the perovskite-type lattice and the local structure around Mn. Results of the analysis for BaTiO_3_:Mn:Bi(0.2%) as a typical example are shown at the bottom of Fig. 3h. This Mn RSF was easily fitted in the *r*-range of 1 to 3 Å with an RSF curve calculated from a model of Mn at the Ti site in BaTiO_3_ that includes appropriate parameters, whereas a model of Mn at the Ba site could not simulate the RSF. Therefore, we confirmed by XAFS analysis that a small amount of Mn occupied the Ti site in BaTiO_3_ in our sample. It has been expected that doped Mn atoms can be substituted for Ti based on indirect data, although there has been hardly any direct evidence^7^,^24^,^25^. Our results provide compelling evidence for this substitution. The analysis suggests the local structures around Mn and Ti are similar, with the analysis of all the RSFs in Fig. 3h indicating that the Mn-O and Mn-Ba bond lengths are slightly shorter than those of Ti-O and Ti-Ba. This difference in bond lengths may explain the local distortion and the reduction of the anisotropy of the tetragonal crystal lattice indicated by the *c/a* ratio shown in Fig. 2b. However, as mentioned above, more convincing and precise data are needed to provide a quantitatively significant description.

### Atomic pair-distribution function (PDF) analysis from high-energy X-ray diffraction

**Figure 4:**
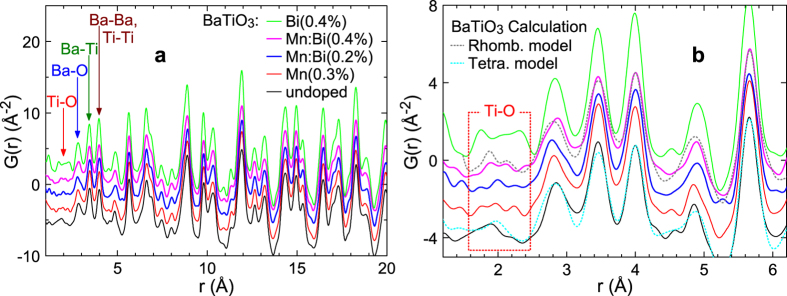
(**a**) Atomic pair-distribution functions of undoped, Bi-doped, and Bi/Mn-doubly doped BaTiO_3_ derived from high-energy X-ray diffraction data. (**b**) Expanded view of Fig. 4a around the peaks related to Ti-O bonds. Calculation results for undoped BaTiO_3_ based on a tetragonal structure model and BaTiO_3_:Mn:Bi(0.4%) based on a rhombohedral structure model are also represented using thin lines in Fig. 4b.

An X-ray diffraction (scattering) pattern at high wavenumbers includes short-range (local) and medium-range structural information. To extract the short-range structural information, we calculated PDFs of undoped, Mn-doped, Bi-doped, and Bi/Mn-doubly doped BaTiO_3_ by Fourier transformation of the corresponding high-energy X-ray diffraction patterns. Figure 4a shows the PDF profiles. These profiles indicate similar structures above a bond length *r* of ~2 Å, and only the shortest Ti-O bonds yielded different shapes for the different samples (Fig. 4b). Although the PDF profile around 2 Å for the undoped BaTiO_3_ can be fitted with a calculation based on a tetragonal structure model, the data for BaTiO_3_:Mn:Bi(0.4%) fits to rather a rhombohedral structure model calculation. As shown in refs 26 and 27, PDF spectrum around 2 Å corresponding to Ti-O bonds in BaTiO_3_ with the tetragonal structure shows a broad single peak and it transforms to a doubly split peak accompanied by a phase transition to the orthorhombic structure and finally the rhombohedral one. The PDF spectra of the undoped BaTiO_3_ and BaTiO_3_:Mn:Bi(0.4%) exhibit the features of tetragonal and rhombohedral structures, respectively. This result means that not only B-site-substituted Mn but also A-site-substituted Bi affected the Ti-O bond, perhaps by affecting the local structure around the B-site atoms with rhombohedral distortion. This may result in the increase of the atomic displacement factor *B*(Ti) by the Rietveld analysis with the tetragonal average structure, as mentioned in the Rietveld analysis subsection and Fig. 2e.

### Estimation of Mn valence from the temperature dependence of magnetic susceptibility

Although a change in Mn valence resulting from the doping of Bi into BaTiO_3_:Mn and BaTiO_3_:Mn:Bi was confirmed by XANES as shown in Fig. 3f, it is difficult to estimate Mn valence quantitatively from the Mn-absorption edge energy compared with reference samples in which the Mn valence is known, such as LaMnO_3_ and SrMnO_3_, because the shapes of the XANES spectra are too different to compare their edge-energies. Many researchers have through the years attempted to estimate the valence of Mn slightly doped in BaTiO_3_. Electron spin resonance (ESR) has been most often used for this purpose^25^,^28^,^29^, because it is very sensitive for the Mn^2+^ ion, but it cannot detect Mn^3+^ and is not very sensitive for Mn^4+^. Therefore, ESR was not suitable for determining the slight change in the Mn valence expected between Mn^4+^ and Mn^3+^ or between Mn^3+^ and Mn^2+^ as a result of Bi-doping. Hagemann *et al*. determined valence states of 3*d*-transition metal elements doped in BaTiO_3_ from the spin states of the dopants from magnetic susceptibility data measured above 77 K^30^. However, this method provided only a rough estimation because it used very small signals detected at high temperatures above 77 K, and used highly doped (up to 2 mol%) materials in which Mn may not have completely dissolved in BaTiO_3_ resulting in some segregation of a secondary phase. Owing to the difficulty of precisely determining the valence of Mn in BaTiO_3_ experimentally, several first-principles studies have been carried out^31^,^32^. Because these calculations may be applicable to a doubly doped BaTiO_3_ system, it would be desirable to use the method to estimate the valence states of Mn in BaTiO_3_:Mn:Bi. Here we attempted to obtain the Mn valence more accurately and precisely not by a first-principles calculation but by an experimental approach, estimating the spin-state of Mn from magnetic susceptibility data acquired more sensitively than in a prior study^30^.

Because the amount of doped Mn is so dilute and the base BaTiO_3_ and the other dopant, Bi, are non-magnetic (diamagnetic), the doped materials should not show any magnetically ordered phase transition even at low temperatures, and the magnetic susceptibility *χ* should obey the Curie-Weiss law. We assumed that the Mn valence is unchanged at temperatures ranging from room temperature to low temperature, because BaTiO_3_:Mn and BaTiO_3_:Mn:Bi showed no significant increase in the dielectric loss relating to electrical conductivity (Fig. 1d), owing to the lack of charge carriers that would have been associated with a valence change. Therefore, magnetic susceptibility was measured down to a low temperature of 2 K; these measurements can be accurate and precise because susceptibility at low temperatures is much larger than at higher temperatures.

**Figure 5:**
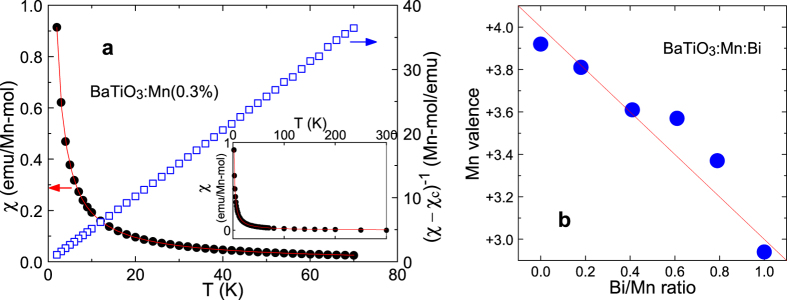
(**a**) Temperature dependence of magnetic susceptibility (*χ*-*T* curve) for BaTiO_3_:Mn(0.3%). Solid line indicates the fit to the Curie-Weiss law with the parameters indicated in Table 2. Inverse susceptibility with subtraction of the constant term (*χ*−*χ*_c_)^−1^ is also indicated. Inset shows the *χ*-*T* curve with a fitted curve in the temperature range of 2 to 300 K. (**b**) Dependence of the Mn valence estimated from the Curie-Weiss analysis of the *χ*−*T* curves on the Bi/Mn content ratio in Mn-doped and Bi/Mn-doubly doped BaTiO_3_.

Figure 5a shows, as an example, the temperature dependence of the magnetic susceptibility (*χ*-*T* curve) of BaTiO_3_:Mn(0.3%). No anomalous magnetic phase transition of the main phase and any secondary phase^33^ was found down to 2 K, which suggests that the susceptibility data can be used in the analysis for estimating the state of Mn. This *χ*-T curve accurately obeys the Curie-Weiss law with an additional assumption of a coexisting component of temperature-independent susceptibility, such as diamagnetic susceptibility^34^, expressed as


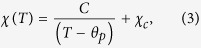


where *C, θ*_*p*_, and *χ*_*c*_ denote the Curie constant, paramagnetic Curie temperature, and temperature-independent susceptibility (constant term), respectively. The relationship between temperature *T* and the inverse of susceptibility minus the constant term, (*χ*−*χ*_*c*_)^−1^, is linear when equation (3) holds. Such linear relationship was found for BaTiO_3_:Mn(0.3%) as seen in Fig. 5a, suggesting that the material containing Mn obeys the Curie-Weiss law very well. Because the magnetic susceptibility obeyed the Curie-Weiss law (3) even at high temperatures up to 300 K, as shown in the inset, the Mn spin state and valence state presumably remained unchanged in the temperature range from room temperature down to 2 K.

Owing to the small signals with large errors at high temperatures and the appearance of a very small difference between field-cooled and zero-field-cooled magnetisations, the *χ*−*T* curves in the temperature range of 2 to 70 K corrected in field-cooled magnetisation were used for a fitting analysis with the Curie-Weiss law (3) to estimate *C, θ*_*p*_, and *χ*_*c*_. Estimated parameters for BaTiO_3_:Mn and BaTiO_3_:Mn:Bi are listed in Table 2 with the reliability factor, *R*^2^, indicating quality of fit, for which a value close to 1 shows that the fitting is reliable. The Curie constant, *C*, is related to Mn spin *S* according to ref. 34


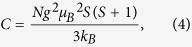


where *N, g, μ*_B_, and *k*_B_ denote the number of Mn ions, the Lande g-factor, the Bohr magneton, and the Boltzmann constant, respectively. The Mn ion in BaTiO_3_ was assumed to have a high-spin state (spin *S* = 5/2, 2, and 3/2 for Mn^2+^, Mn^3+^, and Mn^4+^, respectively), and based on this assumption the obtained *C* was fitted with a linear combination of calculated values of *C* for Mn ions with different valences. Then, the mean valence was derived from the fraction Mn^4+^/Mn^3+^ or Mn^3+^/Mn^2+^, which are listed in Table 2 and plotted in Fig. 5b as a function of the Bi/Mn ratio. The Mn valence decreased monotonically as the Bi/Mn ratio increased. This suggests that doped Mn in BaTiO_3_ was originally Mn^4+^ substituted for Ti^4+^, and Mn^4+^ then received an electron created by the Bi^3+^ substitution for Ba^2+^ so that a corresponding amount of Mn^4+^ was converted to Mn^3+^. The solid red line in Fig. 5b indicates the ideal dependence of Mn valence on Bi/Mn ratio, and the Mn valence estimated from the magnetic susceptibility data almost obeyed this relationship. By this mechanism, Bi can be doped into BaTiO_3_:Mn with stable dielectric properties; in contrast, doping Bi into BaTiO_3_ without Mn degraded the properties with an increase in dielectric loss, caused by an increase in charge carrier density with Bi^3+^ doped at the Ba^2+^ site. The valence of Mn in BaTiO_3_:Mn was estimated to be +3.92, and it is difficult to judge whether the difference between this value and a valence value of +4 is meaningful or within the margin of error. If this difference is meaningful, some of the Mn^4+^ ions may act as acceptors to compensate for intrinsic donors in BaTiO_3_, such as oxygen vacancies, rather than for Bi^3+^ donors, and dielectric loss may have decreased as a result of doping Mn acceptors into BaTiO_3_ (Fig. 1d).

As shown in Table 2, *θ*_*p*_ had a negative and small value, in the order of −0.1 K, indicative of an antiferromagnetic and weak magnetic exchange interaction between Mn spins. This finding is consistent with Mn ions being dispersed thinly in the BaTiO_3_ crystal lattice. The absolute value of *θ*_*p*_ increased with an increasing Bi/Mn ratio, suggesting an increase in the antiferromagnetic exchange interaction between Mn spins due to an increase in the Mn magnetic moment accompanied by a change of the valence of the Mn ion from +4 to +3. The temperature-independent susceptibility *χ*_*c*_ values for BaTiO_3_ and BaTiO_3_:Bi were small negative values, which is typical for diamagnetic behaviour in nonmagnetic insulators, but the *χ*_*c*_ value for BaTiO_3_:Mn:Bi(0.5%) was positive. Furthermore, although the *χ*_*c*_ values for Mn-doped and Bi(0.1–0.4%)/Mn-doubly doped BaTiO_3_ remained negative, their absolute values were smaller than those for BaTiO_3_ and BaTiO_3_:Bi. These results support the existence of a positive component of temperature-independent magnetic susceptibility in this system, and that this positive component was enhanced by Mn doping and Bi/Mn double doping. For example, a magnetically ordered (especially ferromagnetic) impurity might have acted in this way, but the details of this component have not been clarified yet.

## Discussion

As shown in Figs 1e and 2b, doping Bi into BaTiO_3_:Mn stabilised the tetragonal phase with an increase in *T*_C_ and decrease in *T*_ot_, and also increased the *c/a* ratio, the anisotropy of the tetragonal crystal lattice. Two possible reasons for this behaviour should be considered: (i) the effect of the Bi 6 *s*^2^ electron lone pair, or (ii) the Jahn-Teller effect of Mn^3+^ in the MnO_6_ octahedron.

The XAFS analysis revealed the doped Bi atom at the Ba site to be considerably displaced from the centrosymmetric position and the BiO_12_ cluster to be distorted, as shown in Fig. 3d, probably due to the effects of the Bi 6 *s*^2^ electron lone pair. Lattice distortion attributed to the Bi 6 *s*^2^ lone pair has been often reported, for example, in BiMnO_3_^35^ and BiCoO_3_^36^, and Bi displacement from the centrosymmetric position at the A site due to the Bi 6 *s*^2^ lone pair has also been reported in a similar substance, the 0.85BaTiO_3_-0.15BiFeO_3_ solid solution^10^. The tetragonal distortion of PbTiO_3_ has also been attributed to 6 *s*^2^ lone-pair electrons of Pb^2+^ according to the results of a first-principles calculation^37^ and analysis of electron density derived from X-ray diffraction data^38^,^39^. However, Mn^3+^ at the centre of an MnO_6_ octahedron may distort the octahedron and crystal lattice tetragonally, due to the Jahn-Teller effect of the 3*d*^4^ high-spin state, such as in RMnO_3_ (R: rare earth)^40^,^41^ and ZnMn_2_O_4_^40^,^42^. The enhancement of the anisotropy in the tetragonal crystal lattice and the tetragonal phase stabilisation of these samples were confirmed and the mechanism was determined by preparing BaTiO_3_:Mn:(Bi,La) and BaTiO_3_:Mn:La by La^3+^ substitution with no lone-pair electrons for a half or all of the Bi in BaTiO_3_:Mn(0.5%):Bi(0.5%), and then analysing them by X-ray powder diffraction and *ε*_r_−*T* measurement to determine their lattice constants and transition temperatures, respectively.

**Figure 6:**
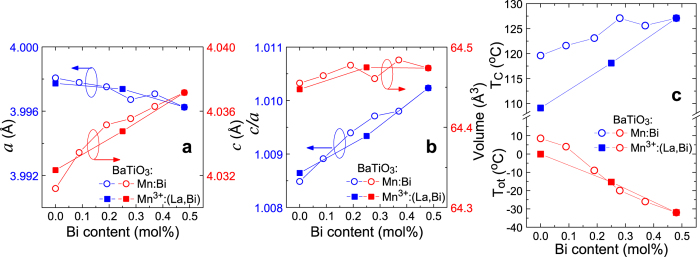
(**a**) Lattice constants *a* and *c* for BaTiO_3_:Mn^3+^:(Bi,La) as a function of Bi content, with those for BaTiO_3_:Mn:Bi shown in Fig. 2a for comparison. (**b**) Lattice volume and the lattice constant *c/a* ratio for BaTiO_3_:Mn^3+^:(Bi,La) as a function of Bi content, with those for BaTiO_3_:Mn:Bi shown in Fig. 2b for comparison. (**c**) *T*_C_ and *T*_ot_ for BaTiO_3_:Mn^3+^:(Bi,La) as a function of Bi content, with those for only BaTiO_3_:Mn:Bi shown in Fig. 1e for comparison.

Figure 6a and b show the lattice constants *a* and *c*, the *c/a* ratio, and unit cell volume for BaTiO_3_:Mn^3+^:Bi, BaTiO_3_:Mn^3+^:(Bi,La), and BaTiO_3_:Mn^3+^:La, as a function of Bi content, compared with those for BaTiO_3_:Mn:Bi, in which Mn valence depends on Bi content (detailed data are available in Supplementary Information). The dependence of the lattice parameters on Bi content for BaTiO_3_:Mn^3+^:(Bi,La) was the same as that for BaTiO_3_:Mn:Bi, even though the Mn valence was always +3 independently of the Bi content. The dependence of the transition temperatures *T*_C_ and *T*_ot_ on Bi content for BaTiO_3_:Mn^3+^:(Bi,La) were similar to those for BaTiO_3_:Mn:Bi, as shown in Fig. 6c. These results indicate that the tetragonality enhancement and the tetragonal phase stabilisation should be attributed not to the Mn^3+^ fraction but to the Bi content. Therefore, we conclude that local distortion by Bi 6 *s*^2^ lone pair electrons enhances the tetragonal anisotropy and stabilises the tetragonal phase in BaTiO_3_:Mn:Bi.

## Conclusions

In summary, enhancement of the anisotropy of the tetragonal crystal lattice and the tetragonal phase stabilisation of Bi/Mn-doubly doped BaTiO_3_ ceramics were investigated. XAFS measurements confirmed that Bi and Mn were located at the A site (Ba site) and B site (Ti site), respectively. Powder X-ray diffraction revealed the tetragonal crystal lattice constants *a* and *c* shrank and elongated, respectively, and hence the ratio *c/a* increased with increasing Bi content, although with no change in the unit cell volume. Moreover, *T*_C_ lowered by Mn-doping was increased to recover and *T*_ot_ was decreased by doping very small amounts of Bi (up to 0.5 mol%) into BaTiO_3_:Mn. Analysis of Bi-*L*_3_ EXAFS clarified the presence of a considerable deviation of the Bi position from the centrosymmetric position, and its resulting distortion of the BiO_12_ cluster in Bi-doped and Bi/Mn-doubly doped BaTiO_3_. PDF and Rietveld analyses also revealed that doped Bi at the A site caused local structure distortions around the B-site atoms. The Mn valence was evaluated from its spin state estimated by magnetic susceptibility measurements, revealing a change of the Mn valence from +4 to +3 upon addition of the same molar amount of Bi as Mn, probably due to the compensating behaviour of the Mn that originally has a valence state of +4 at Ti^4+^ sites for donor doping of Bi^3+^ into the Ba^2+^ site. Based on the Bi-*L*_3_ EXAFS results and the observation that adding La^3+^ instead of Bi^3+^ did not increase the *c/a* ratio or increase *T*_C_ and decrease *T*_ot_, the enhancement of the anisotropy in the tetragonal crystal lattice and stabilisation of the tetragonal phase by adding Bi in BaTiO_3_:Mn appeared to be caused by the effect of the 6 *s* lone-pair electrons in Bi^3+^ at the Ba site in BaTiO_3_, not by the Jahn-Teller effect of Mn^3+^ in the MnO_6_ octahedron, similar to Pb^2+^ in tetragonally distorted PbTiO_3_.

## Methods

### Sample preparation

BaTiO_3_, BaTiO_3_-0.004BiO_3/2_, Ba(Ti_0.995_Mn_0.005_)O_3_-xBiO_3/2_ (x = 0.001, 0.002, 0.003, 0.004, and 0.005), Ba(Ti_0.995_Mn_0.005_)O_3_, Ba(Ti_0.997_Mn_0.003_)O_3_, Ba(Ti_0.995_Mn_0.005_)O_3_-0.005LaO_3/2_, and Ba(Ti_0.995_Mn_0.005_)O_3_-0.005(La_0.5_Bi_0.5_)O_3/2_ ceramic samples denoted, respectively, as undoped BaTiO_3_, BaTiO_3_:Bi, BaTiO_3_:Mn:Bi(0.1, 0.2, 0.3, 0.4, 0.5%), BaTiO_3_:Mn(0.3, 0.5%), BaTiO_3_:Mn:La, and BaTiO_3_:Mn:(La,Bi) were prepared by conventional solid state synthesis and sintering. Submicrometre-sized BaTiO_3_ powder (BT01, Sakai Chemical Industry) and stoichiometric amounts of micrometre-sized Bi_2_O_3_ (99.999%, Kojundo Chemical Laboratory), MnO_2_ (99.99%, Kojundo), BaCO_3_ (99.95%, Kojundo), and La_2_O_3_ (99.999%, Kojundo, dehydrated by heating at 900 °C before weighing) powders were weighed and mixed in a planetary ball mill with zirconia beads (1 and 3 mm in diameter) and ethanol at a rotation speed of 500 rpm for 12 h to homogenise. An ethanol solution of polyvinyl butyral was added to the mixed powders as a binder, they were mixed well and dried, and then the powders were pressed uniaxially into pellets 17 mm in diameter. The pellets were fired at 1350 °C for 4 h in a conventional box furnace with molybdenum disilicide heaters. The surfaces of the ceramic disks were ground slightly to remove surface layers that might have been contaminated during the firing process. The amounts of Bi and Mn additives in the ceramics were checked by using inductively coupled plasma mass spectroscopy and atomic emission spectroscopy, respectively (data are shown in Supplementary Information). All plots with respect to Bi content or Bi/Mn content ratio used the measured amounts of Bi/Mn, although the nominal values were used in the chemical formula. Homogeneity of the samples were checked by scanning electron microscopy and transmission electron microscopy, and discussed using the data of electrical characteristics, x-ray diffraction, XAFS, and magnetic measurements. The details are given in Supplementary Information.

### Dielectric and ferroelectric characterisations

For electrical characterisation, the ceramic disks were polished to a thickness of about 0.5 mm. Gold films (300 nm thick) with titanium glue layers (30 nm) were deposited on both polished sides of each sample as electrodes by magnetron sputtering in an argon atmosphere. Then, the samples were cut into 2.5 × 10.0 mm pieces with a dicing saw. The temperature dependence of the dielectric permittivity and loss factor were measured with frequencies of 1, 10, and 100 kHz under ambient conditions by using an impedance analyser (IM3570, Hioki) and a temperature-controlled stage system (LTS350, Linkam). Polarisation-electric field (*P*–*E*) curves were characterised with a ferroelectric characteristics evaluation system (FCE-1, Toyo) at room temperature.

### Synchrotron X-ray powder diffraction and Rietveld analysis

X-ray diffraction of BaTiO_3_:Mn(0.3%), BaTiO_3_:Bi, BaTiO_3_:Mn:Bi, BaTiO_3_:Mn:La, and BaTiO_3_:Mn:(La,Bi) powders was carried out at room temperature with a large-scale Debye-Scherrer camera^43^ at the BL19B2 bending-magnet beamline of SPring-8^44^. The incident X-ray beam was monochromated to a wavelength λ = 0.39984 or 0.39987 Å. Pure sample powder and sample powder well mixed with CeO_2_ standard material (NIST 640a) were prepared for each substance for more accurate estimation of lattice constants. Data were recorded on an imaging plate serving as a two-dimensional detector with a step interval of 0.01°.

The Rietveld method was used to analyse the powder diffraction data with Rietan-FP^45^ software with the VESTA^46^ program for visualising and analysing crystal structures. First, a Rietveld analysis for determining lattice constants was performed with data taken from the mixed specimen with the standard. Then, other lattice parameters (atomic positions and displacement factors) were successively derived from data taken from the pure powder sample using the lattice constants derived from the Rietveld analysis with the data taken from the powder sample mixed with the standard (see Supplementary Information).

### XAFS

X-ray absorption spectra of Bi-*L*_3_, Ba-*K*, and Mn-*K* were collected at the BL14B2 bending magnet beamline of SPring-8^47^. To control the wavelength (energy) of the incident X-ray beam, a double-crystal monochromator with Si(311) for Bi-*L*_3_ and Ba-*K*, and with Si(111) for Mn-*K* XAFS measurements was employed. The fluorescent method with an array of 19 elements of Ge solid-state detectors was used for Bi-*L*_3_ and Mn-*K* XAFS measurements for BaTiO_3_:Mn(0.5%), BaTiO_3_:Bi, and BaTiO_3_:Mn:Bi samples because Bi and Mn were dilute, whereas Ba-*K* XAFS spectra were taken by the transmission method with an ionisation chamber with samples diluted with boron nitride (BN) powder. The intensity of the incident X-ray beam was monitored with another ionisation chamber in both fluorescence and transmission modes. As standard samples, BaBiO_3_, BiFeO_3_, and 0.67BiFeO_3_-0.33BaTiO_3_ solid solution^48^ were prepared by a conventional solid-state reaction method for Bi-*L*_3_ XAFS, and commercially available LaMnO_3_ (99.9%, Toshima Manufacturing) and SrMnO_3_ (99.9%, Toshima Manufacturing) were used for Mn-*K* XAFS analysis. XAFS measurements of the standard samples were taken by using the transmission method with BN-diluted disk-samples. The collected data were analysed with ATHENA/ARTEMIS software programs^49^ in which FEFF6 code^21^ was used to calculate scattering paths. The Fourier transformation of the Bi-*L*_3_ EXAFS profile, *k*^3^*χ(k*), to calculate the Bi RSF was performed with *k* from 2.5 to 9.5 Å^−1^, and curve fitting of the Bi RSF with single scattering EXAFS equation (2) was performed in an *r* space of 1.1 to 4.3 Å, in which no multiple scattering path exists in the (Ba,Bi)TiO_3_ model. In addition, the Fourier transformation of the Ba-*K* EXAFS profile, *k*^3^*χ(k*), to derive the Ba RSF was performed with *k* from 3.0 to 12.0 Å^−1^, and that of the Mn-*K* EXAFS profile, *k*^2^*χ(k*), to derive the Mn RSF was performed with *k* from 2.0 to 7.0 Å^−1^. The fitted curve of the Mn RSF was calculated in an *r* space of 1.0 to 3.5 Å.

### High-energy powder x-ray diffraction and PDF analysis

High-energy (60 keV) X-ray diffraction experiments for PDF analysis were carried out at the BL14B1 bending-magnet beamline at SPring-8^5^^0^. A kapton (polyimide) capillary tube was filled with the powder sample. A Ge point detector was scanned to collect the scattered X-rays from the sample up to a wavenumber *Q* = 17 Å^−1^. The data were corrected for background, absorption, multiple-scattering, and inelastic effects, and then were normalised to the incident flux and the total sample scattering cross section to yield the total scattering structure function, *S(Q*). The reduced PDF, *G(r*), was derived by Fourier transformation of *S(Q*). The obtained PDF profiles were analysed calculations using the program PDFgui^51^.

### Magnetic susceptibility measurements

The temperature dependence of magnetisation was measured by using a superconducting quantum interference device DC magnetometer (MPMS-7/XL7, Quantum Design) with a temperature range of 2–300 K under a DC magnetic field of 100 Oe. A linear dependence of the magnetisation on the magnetic field up to 100 Oe was confirmed for every sample and in the entire temperature range, so magnetic susceptibility could be derived easily from the magnetisation values divided by the magnitude of the magnetic field. A gelatine capsule was filled with the accurately weighted powder sample (~200 mg) and mounted in the magnetometer with a plastic straw sample holder. DC mode was used for the measurements and the scan length was set to 5 cm. A Pd reference sample (Quantum Design) was also used to calibrate the magnetisation value of the machine each time. The temperature dependence of the magnetisation was determined under both zero-field cooling and field cooling conditions. To estimate the magnetic moment of Mn in the sample, susceptibility measured with field cooling at 2–70 K was extracted and fitted according to the Curie-Weiss law.

## Additional Information

**How to cite this article:** Yabuta, H. *et al*. Enhancement of tetragonal anisotropy and stabilisation of the tetragonal phase by Bi/Mn-double-doping in BaTiO_3_ ferroelectric ceramics. *Sci. Rep.* **7**, 45842; doi: 10.1038/srep45842 (2017).

**Publisher's note:** Springer Nature remains neutral with regard to jurisdictional claims in published maps and institutional affiliations.

## Supplementary Material

Supplementary Information

## Figures and Tables

**Table 1:** Fitting results for the radial distribution functions for BaTiO_3_:Mn:Bi(0.4%) and BaTiO_3_:Bi(0.4%) from the Bi-*L*_3_ EXAFS spectra shown in Fig. 3c.

	Bi-O	Bi-Ti	Bi-Ba
BaTiO_3_:Mn:Bi(0.4%) (R = 0.00009)	4 × 2.254	4 × 3.295	1 × 3.834
	4 × 2.738	4 × 3.698	4 × 3.996
	4 × 3.045		1 × 4.499
BaTiO_3_:Bi(0.4%) (R = 0.00009)	4 × 2.258	4 × 3.298	1 × 3.978
	4 × 2.757	4 × 3.704	4 × 3.775
	4 × 3.088		1 × 4.424

**Table 2:** Results of the Curie-Weiss analysis of *χ*−*T* curves for Mn-doped and Bi/Mn-doubly doped BaTiO_3_.

	*C* (emuK/Mn-mol)	**Mn valence**	*χ*_c_ (10^−6^ emu/mol)	*θ*_p_ (K)	*R*^2^
BaTiO_3_:Mn(0.3%)	1.963	**+3.92**	−7.79	−0.14	0.99998
BaTiO_3_:Mn:Bi(0.1%)	2.085	**+3.81**	−2.12	−0.45	0.99996
BaTiO_3_:Mn:Bi(0.2%)	2.317	**+3.61**	−2.30	−0.40	0.99991
BaTiO_3_:Mn:Bi(0.3%)	2.362	**+3.57**	−2.30	−0.58	0.99975
BaTiO_3_:Mn:Bi(0.4%)	2.584	**+3.37**	−2.35	−0.73	0.99960
BaTiO_3_:Mn:Bi(0.5%)	3.068	**+2.94**	+10.1	−0.76	0.99983
BaTiO_3_^a^			−21.4^a^		
BaTiO_3_:Bi(0.4%)^a^			−18.7^a^		

^a^For undoped BaTiO_3_ and BaTiO_3_;Bi, diamagnetic susceptibility measured at 300 K is shown.
