# Supplementary material for: Enhancement of tetragonal anisotropy and stabilisation of the tetragonal phase by Bi/Mn-double-doping in BaTiO3 ferroelectric ceramics
Source: Sci Rep. 2017 Apr 3;7:45842. doi: 10.1038/srep45842 (PMC5377453; doi:10.1038/srep45842)
Supplement: Supplementary Information [file srep45842-s1.pdf]

Supplementary Information for
"Enhancement of tetragonal anisotropy and stabilisation of the tetragonal phase by Bi/Mn-double-doping in $BaTiO_3$ ferroelectric ceramics"

Hisato Yabuta,* Hidenori Tanaka, Tatsuo Furuta, Takayuki Watanabe,
Makoto Kubota, Takanori Matsuda, and Toshihiro Ifuku
R&D Headquarters, Canon Inc.,
Ohta, Tokyo 146-8501, Japan

Yasuhiro Yoneda
Reaction Dynamics Research Center, Japan Atomic Energy Agency (JAEA), Sayo-cho, Hyogo 679-5148, Japan

I. ADDITIVE ELEMENT CONTENTS IN THE SAMPLES

The amounts of Bi and Mn in $BaTiO_3$:Bi, $BaTiO_3$:Mn, $BaTiO_3$:Mn:Bi samples were measured by inductively coupled plasma mass spectroscopy (ICP-MS) and atomic emission spectroscopy (ICP-AES), respectively. Mn and Bi contents calculated from the measured amounts are shown in TABLE SI. Amounts of La, Bi, and Mn additives in $BaTiO_3$:Mn:La and $BaTiO_3$:Mn:(Bi,La) samples have not been measured by ICP-MS or ICP-AES so that the nominal values of the additives for these samples are indicated in the figure.

TABLE SI. Mn and Bi contents by means of ICP-AES and ICP-MS, respectively, for pristine $BaTiO_3$, $BaTiO_3$:Bi, $BaTiO_3$:Mn, and $BaTiO_3$:Mn:Bi. Nominal values of Mn, Bi, and La contents in $BaTiO_3$:Mn:La, and $BaTiO_3$:Mn:(Bi,La) samples are also indicated with an Italic font.

Sample	Abbreviation	Mn(mol%)	Bi(mol%)	La(mol%)
$BaTiO_3$	BT	–	–	–
$BaTiO_3$:Bi(0.4%)	BTB	–	0.37	–
$BaTiO_3$:Mn(0.3%)	BTM03	0.33	–	–
$BaTiO_3$:Mn(0.5%)	BTM05	0.47	–	–
$BaTiO_3$:Mn:Bi(0.1%)	BTMB01	0.49	0.09	–
$BaTiO_3$:Mn:Bi(0.2%)	BTMB02	0.46	0.19	–
$BaTiO_3$:Mn:Bi(0.3%)	BTMB03	0.46	0.28	–
$BaTiO_3$:Mn:Bi(0.4%)	BTMB04	0.47	0.37	–
$BaTiO_3$:Mn:Bi(0.5%)	BTMB05	0.48	0.48	–
$BaTiO_3$:Mn:(Bi,La)	BTMBL	*0.5*[a]	*0.25*[a]	*0.25*[a]
$BaTiO_3$:Mn:La	BTML	*0.5*[a]	–	*0.5*[a]

[a] Nominal composition.

*yabuta.hisato@canon.co.jp

II. HOMOGENEITY OF THE SAMPLES

A. Electron microscopy

Before this study, we observed secondary phases containing considerable amount of Mn at triple junctions of grains in relatively highly Mn-doped ($\gtrsim$1 mol%) $BaTiO_3$ samples by scanning electron microscopy (SEM) and transmission electron microscopy (TEM). The samples were prepared with raw powders mixed in an agate mortar by hand. Therefore, doping amount into $BaTiO_3$ was set to 0.5 mol% or less and a planetary ball mill was used for the raw powder mixing process in order to obtain homogenised samples. No secondary phase was observed in the samples by SEM and TEM.

B. Magnetic susceptibility

Since the Mn-contained secondary phases showed a magnetic order at low temperatures, temperature dependence of magnetic susceptibility for the highly Mn-doped $BaTiO_3$ samples deviated slightly from the Curie-Weiss law. In contrast, magnetic susceptibility of the samples in this study obeyed the Curie-Weiss law very well ($R^2 > 0.9995$) as indicated in Table 2. This suggests that formation of Mn-contained secondary phases is negligible.

C. X-ray powder diffraction

Even though the raw powders were well mixed using a planetary ball mill, diffraction peaks from undefined impurity phases were detected for the undoped and doped $BaTiO_3$ samples. However, no specific peak attributed to Mn- and/or Bi-doping was clearly found, and the intensity ratio of the largest unidentified peak to the largest peak from the main phase for the undoped $BaTiO_3$ (about 6:1000) was decreased to about 1:1000 by Mn- and/or Bi-doping. These are indicative of no segregated secondary phase directly accompanied by the Mn- and/or Bi-doping.

D. XAFS

As indicated in Fig. 3c and Table 1, Bi-L_3 EXAFS spectra were well fitted with the single coordination model of Bi at A-site in the perovskite-type $BaTiO_3$. This suggests that Bi atoms existed almost in $BaTiO_3$ and those in secondary phases with other coordination should be rare.

E. Temperature dependence of permittivity

Although T_C shifted by Mn- and/or Bi-doping, abruptness of the permittivity change at T_C almost unchanged. This means that the dopants were distributed in the host $BaTiO_3$ homogeneously, because the permittivity change at T_C would be gradual if concentration of the dopants were fluctuated.

F. Impedance (dielectric) spectroscopy

The dielectric permittivity data shown in Figs. 1a-d were derived from impedance measurements. The relation between the complex permittivity $\varepsilon^* = \varepsilon' + j\varepsilon''$ (ε', ε'': real and imaginary parts of permittivity, respectively) and the complex impedance $Z^* = Z' + jZ''$ (Z', Z'': real and imaginary parts of impedance, respectively) are given by

$$\varepsilon^* = \{2\pi f(\varepsilon_0 A/l)jZ^*\}^{-1} \tag{S1}$$

where f: frequency, A: sample area, and l: sample thickness. For electrical heterogeneous samples with serial connections of parallel resistance-capacitance (R-C) components (e.g., a serial connection of grain bulk and grain boundary), the total impedance is given by

$$Z^* = \sum_i \frac{R_i}{1 + 2\pi f j R_i C_i} \tag{S2}$$

where R_i, C_i represents resistance and capacitance of ith component, respectively, and also imaginary part of impedance are written as

$$Z'' = \sum_i \frac{R_i(2\pi f R_i C_i)}{1+(2\pi f R_i C_i)^2}. \tag{S3}$$

In a frequency range of 10^2-10^7 Hz, Z'' for undoped, Mn-doped, and Bi/Mn-doubly doped $BaTiO_3$ at room temperature obeyed well a relation of $d\log Z''/d\log f = -1$, and also frequency dependence of ε' for these samples showed almost constant. This means that these samples electrically consist of only one component with a large $RC \gg (2\pi f)^{-1}$, suggesting homogeneous capacitors (insulators).
In contrast, impedance of Bi-doped $BaTiO_3$ showed relatively conductive behaviour consisting of two electrical components, bulk (b) and grain boundary (gb). Each resistance (R_b, R_{gb}) and capacitance (C_b, C_{gb}) were roughly estimated from Z^* and the complex electric modulus $M^* = M' + jM'' = 1/\varepsilon^*$ of $BaTiO_3$:Bi(0.4%) at room temperature: $R_b \simeq 10^4$ Ω, $R_{gb} \simeq 10^1$ Ω, $C_b \simeq C_{gb} = 10^{-9} - 10^{-10}$ F. The huge dielectric loss of $BaTiO_3$:Bi shown in Fig. 1d are owing to these row resistances, especially low R_{gb}, which may not be caused by segregation of Bi-contained conductive layer at the grain boundaries, taking the XAFS result into account, but lowering of potential barrier height at the interfaces of Bi-doped bulk grains.

Considering the data and the discussions above mentioned, the doped $BaTiO_3$ samples in this study are homogeneous enough to be employed for the measurements and the analyses.

III. X-RAY POWDER DIFFRACTION AND RIETVELD ANALYSIS

As described in METHODS section, X-ray powder diffraction was carried out twice with a mixed powder specimen with the CeO_2 standard and a pure one for each sample. Diffraction patterns taken with the mixed and pure specimens of $BaTiO_3$:Mn:Bi(0.2%) as a representative of the samples in this study are shown in Figs. S1 and S2, respectively. First, a diffraction pattern of the mixed specimen was refined by the two-phase Rietveld method with the fixed lattice constant of the CeO_2 standard, as shown in Fig. S1. Secondary, using the obtained lattice constants a and c from this refinement as fixed parameters, the single-phase Rietveld analysis for a diffraction pattern of the pure specimen was carried out, as shown in Fig. S2, in order to obtain c-axis atomic position z and isotropic atomic displacement factor B for each atom. Obtained crystallographic parameters for the samples in this study by the first and the second Rietveld analyses are listed in Tables SII and SIII, respectively. Some criteria of fit[1] for each refinement are also indicated in the tables.

[1] R. A. Young, in *The Rietveld Method*, IUCr Monograph on Crystallography, Vol. 5, edited by R. A. Young (Oxford University Press, Oxford, 1993), Chap 1, pp. 1–38.

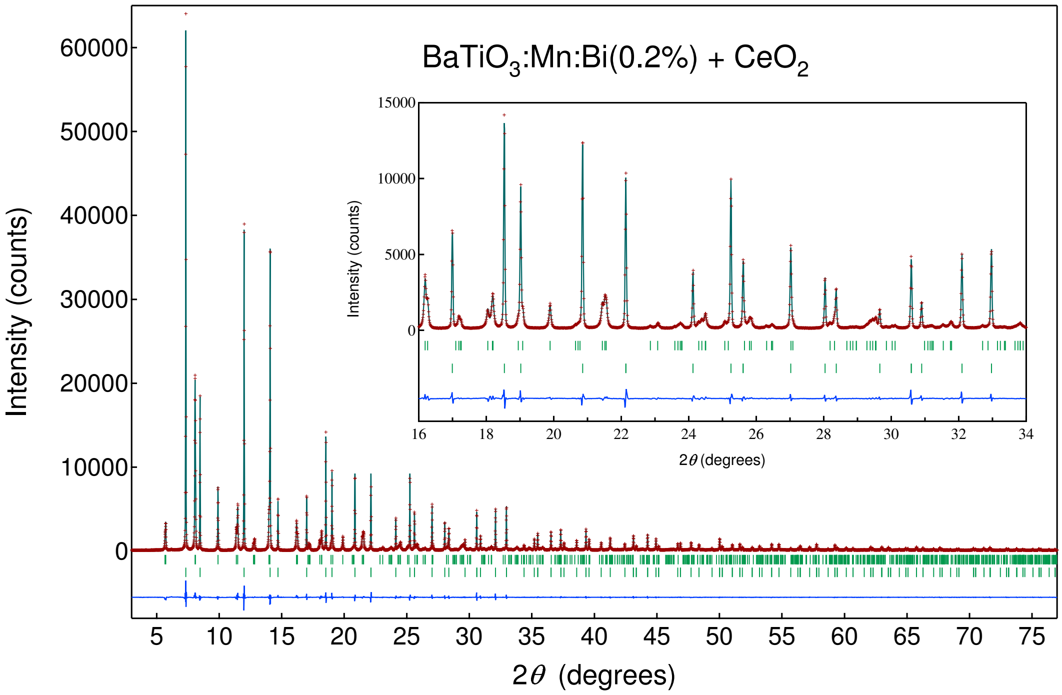

FIG. S1. Observed X-ray diffraction pattern (red crosses) and the result of Rietveld analysis (green line) for mixed powder specimen of $BaTiO_3$:Mn:Bi(0.2%) with the CeO_2 standard. Green ticks below the pattern indicate Bragg peak positions of $BaTiO_3$:Mn:Bi(0.2%) (upper) and CeO_2 (lower). Blue line at the bottom denotes the difference between the observed pattern and the fitting result. The inset shows an expanded view of the patterns in the 2θ range of 16° to 34°.

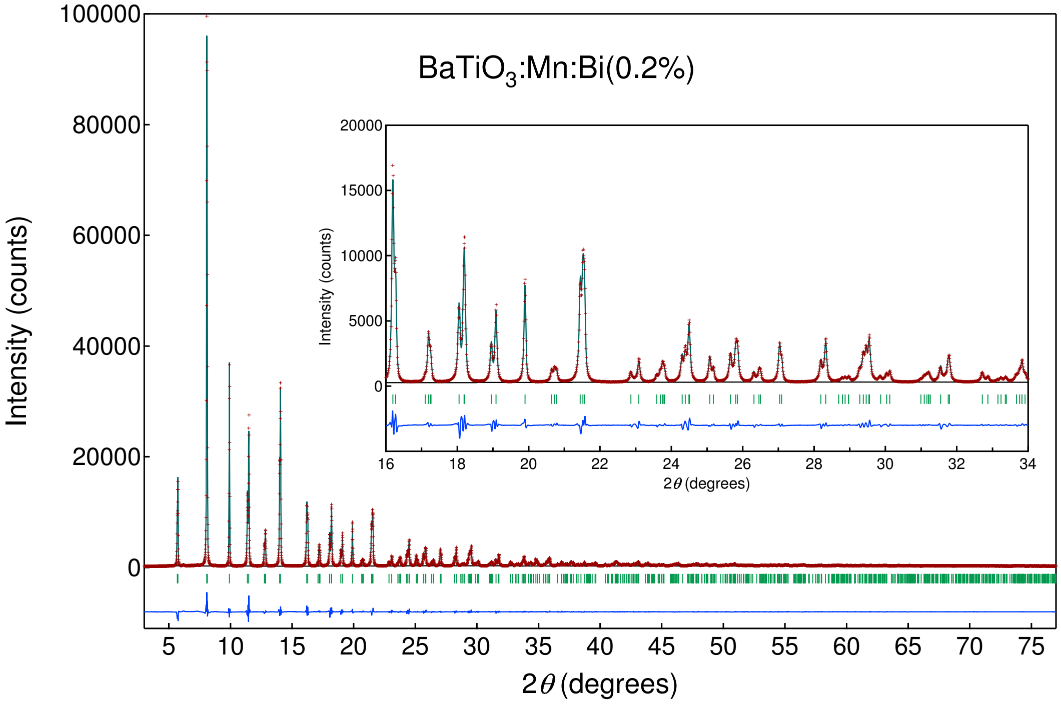

FIG. S2. Observed X-ray diffraction pattern (red crosses) and the result of Rietveld analysis (green line) for pure powder specimen of $BaTiO_3$:Mn:Bi(0.2%). Green ticks below the pattern indicate Bragg peak positions of $BaTiO_3$:Mn:Bi(0.2%). Blue line at the bottom denotes the difference between the observed pattern and the fitting result. The inset shows an expanded view of the patterns in the 2θ range of 16° to 34°.

TABLE SII. Refined crystallographic parameters from the Rietveld analysis for mixed powder specimens of pristine $BaTiO_3$, $BaTiO_3$:Bi, $BaTiO_3$:Mn, $BaTiO_3$:Mn:Bi, $BaTiO_3$:Mn:La, and $BaTiO_3$:Mn:(Bi,La) samples with the CeO_2 standard for obtaining reliable values of the lattice parameters a and c, which are indicated with a bold font. Fraction ratio between the $BaTiO_3$-based sample and the CeO_2 standard obtained from the fitting and some criteria of fit [1], R_{wp} (R-wieghted pattern), R_{B} (R-Bragg factor), R_{F} (R-structure factor) and S (*Goodness-of-fit*), are also represented in the table.

Sample (abbr.)		BT	BTB	BTM03	BTMB01	BTMB02	BTMB03	BTMB04	BTMB05	BTMBL	BTML
	$\boldsymbol{a}$(Å)	**3.99564**(6)	**3.99743**(3)	**3.99807**(3)	**3.99779**(5)	**3.99752**(7)	**3.99673**(5)	**3.99708**(5)	**3.99625**(4)	**3.99738**(4)	**3.99773**(4)
	$\boldsymbol{c}$(Å)	**4.03279**(7)	**4.03441**(5)	**4.03199**(6)	**4.03344**(6)	**4.03515**(8)	**4.03555**(6)	**4.03630**(6)	**4.03716**(5)	**4.03475**(5)	**4.03233**(4)
Ba:	B(Å^2)	0.237(7)	0.313(7)	0.254(7)	0.269(7)	0.301(9)	0.285(7)	0.301(8)	0.331(6)	0.300(6)	0.304(5)
Ti:	z	0.0180(9)	0.0151(14)	0.0117(19)	0.0155(10)	0.013(2)	0.0150(11)	0.0152(12)	0.0131(11)	0.0134(10)	0.0121(11)
	B(Å^2)	0.25(2)	0.32(2)	0.31(2)	0.25(14)	0.29(2)	0.29(2)	0.29(2)	0.375(16)	0.339(14)	0.372(14)
O(1):	z	0.470(2)	0.467(3)	0.462(3)	0.468(2)	0.465(3)	0.469(2)	0.467(2)	0.468(2)	0.469(2)	0.471(2)
	B(Å^2)	0.14(11)	0.24(15)	0.4(2)	0.14(11)	0.16(14)	0.21(11)	0.12(11)	0.35(11)	0.04(8)	0.09(8)
O(2):	z	-0.005(5)	-0.008(7)	-0.010(6)	-0.009(5)	-0.007(8)	-0.007(5)	-0.006(6)	-0.010(5)	-0.012 (4)	-0.009(5)
	B(Å^2)	0.42(5)	0.35(7)	0.19(8)	0.38(6)	0.43(8)	0.39(6)	0.41(6)	0.42(6)	0.47(6)	0.52(5)
	R_{wp}	5.024	5.473	6.026	5.136	5.131	5.374	4.995	5.523	5.185	5.516
	S	1.0692	1.3155	1.3037	1.1629	1.1031	1.2795	1.1036	1.4319	1.3299	1.4901
	Fraction	0.6779	0.4378	0.5345	0.6556	0.446	0.6362	0.5172	0.6644	0.7711	0.773
$BaTiO_3$:	R_{B}	1.056	1.125	1.026	1.238	1.171	1.243	1.469	1.271	1.015	1.228
	R_{F}	0.685	1.008	0.723	0.843	0.809	0.833	0.999	0.854	0.59	0.906
CeO_2:	R_{B}	1.113	1.176	0.934	1.144	1.045	1.287	1.265	1.264	0.824	1.101
	R_{F}	0.925	1.142	0.896	0.999	0.976	0.976	1.143	0.936	0.629	1.02

TABLE SIII. Refined crystallographic parameters from the Rietveld analysis for pure powder specimens of pristine $BaTiO_3$, $BaTiO_3$:Bi, $BaTiO_3$:Mn, $BaTiO_3$:Mn:Bi, $BaTiO_3$:Mn:La, and $BaTiO_3$:Mn:(Bi,La) samples with a and c fixed to the values in TABLE SII (indicated with an Italic font in the table) for obtaining reliable values of z (c-axis atomic position) and B (atomic displacement factor), which are indicated with a bold font. Some criteria of fit [1], R_{wp} (R-wieghted pattern), R_{B} (R-Bragg factor), R_{F} (R-structure factor) and S (*Goodness-of-fit*), are also represented in the table.

Sample (abbr.)		BT	BTB	BTM03	BTMB01	BTMB02	BTMB03	BTMB04	BTMB05	BTMBL	BTML
	a(Å)	*3.99564*[a]	*3.99743*[a]	*3.99807*[a]	*3.99779*[a]	*3.99752*[a]	*3.99673*[a]	*3.99708*[a]	*3.99625*[a]	*3.99738*[a]	*3.99773*[a]
	c(Å)	*4.03279*[a]	*4.03441*[a]	*4.03199*[a]	*4.03344*[a]	*4.03515*[a]	*4.03555*[a]	*4.03630*[a]	*4.03716*[a]	*4.03475*[a]	*4.03233*[a]
Ba:	$\boldsymbol{B}$(Å^2)	**0.253**(6)	**0.289**(4)	**0.262**(5)	**0.288**(5)	**0.279**(4)	**0.316**(5)	**0.295**(4)	**0.324**(4)	**0.307**(4)	**0.301**(4)
Ti:	$\boldsymbol{z}$	**0.0169**(9)	**0.0129**(8)	**0.0151**(9)	**0.0149**(9)	**0.0142**(8)	**0.0127**(9)	**0.0123**(9)	**0.0133**(8)	**0.0128**(8)	**0.0128**(8)
	$\boldsymbol{B}$(Å^2)	**0.31**(2)	**0.374**(11)	**0.308**(14)	**0.323**(14)	**0.337**(12)	**0.392**(12)	**0.373**(11)	**0.384**(11)	**0.381**(11)	**0.370**(11)
O(1):	$\boldsymbol{z}$	**0.471**(2)	**0.473**(2)	**0.474**(2)	**0.475**(2)	**0.472**(2)	**0.474**(2)	**0.471**(2)	**0.471**(2)	**0.474**(2)	**0.475**(2)
	$\boldsymbol{B}$(Å^2)	**0.31**(11)	**0.32**(7)	**0.32**(10)	**0.06**(8)	**0.26**(8)	**0.09**(7)	**0.25**(7)	**0.41**(8)	**0.14**(7)	**0.12**(7)
O(2):	$\boldsymbol{z}$	**-0.010**(4)	**-0.010**(3)	**-0.010**(4)	**-0.009**(4)	**-0.010**(3)	**-0.007**(5)	**-0.008**(4)	**-0.011**(3)	**-0.010** (3)	**-0.009**(4)
	$\boldsymbol{B}$(Å^2)	**0.36**(6)	**0.40**(4)	**0.38**(5)	**0.59**(6)	**0.40**(4)	**0.58**(5)	**0.45**(4)	**0.36**(4)	**0.53**(4)	**0.55**(4)
	R_{wp}	7.160	5.756	6.336	6.506	5.510	6.302	5.911	5.813	5.708	5.766
	S	1.8509	1.4001	1.9065	1.7736	1.5846	1.7706	1.8146	1.5822	1.5633	1.6119
$BaTiO_3$:	R_{B}	1.257	1.078	1.102	1.078	1.111	1.143	1.152	1.021	1.027	1.148
	R_{F}	0.515	0.497	0.462	0.401	0.600	0.575	0.583	0.601	0.477	0.463

[a] Fixed.
